# New risk score of the early period after spontaneous subarachnoid hemorrhage: For the prediction of delayed cerebral ischemia

**DOI:** 10.1111/cns.13202

**Published:** 2019-08-12

**Authors:** Yuan‐Jian Fang, Shu‐Hao Mei, Jia‐Nan Lu, Yi‐Ke Chen, Zhao‐Hui Chai, Xiao Dong, Camila Araujo, Cesar Reis, Jian‐Min Zhang, Sheng Chen

**Affiliations:** ^1^ Department of Neurosurgery, The Second Affiliated Hospital, School of Medicine Zhejiang University Hangzhou China; ^2^ Department of Neurosurgery, The First Affiliated Hospital, School of Medicine Zhejiang University Hangzhou China; ^3^ Department of Physiology and Pharmacology Loma Linda University School of Medicine Loma Linda CA USA; ^4^ Brain Research Institute Zhejiang University Hangzhou China; ^5^ Collaborative Innovation Center for Brain Science Zhejiang University Hangzhou China

**Keywords:** delayed cerebral ischemia, early brain injury, grading system, intracranial aneurysm, subarachnoid hemorrhage

## Abstract

**Background and Purpose:**

The aim of this study is to identify the early predictors for delayed cerebral ischemia (DCI) and develop a risk stratification score by focusing on the early change after aneurysmal subarachnoid hemorrhage (aSAH).

**Methods:**

The study retrospectively reviewed aSAH patients between 2014 and 2015. Risk factors within 72 hours after aSAH were included into univariable and multivariable logistic regression analysis to screen the independent predictors for DCI and to design a risk stratification score.

**Results:**

We analyzed 702 aSAH patients; four predictors were retained from the final multivariable analysis: World Federation of Neurosurgical Societies scale (WFNS; OR = 4.057, *P* < .001), modified Fisher Scale (mFS; OR = 2.623, *P* < .001), Subarachnoid Hemorrhage Early Brain Edema Score (SEBES; OR = 1.539, *P* = .036), and intraventricular hemorrhage (IVH; OR = 1.932, *P* = .002). According to the regression coefficient, we created a risk stratification score ranging from 0 to 7 (WFNS = 3, mFS = 2, SEBES = 1, and IVH = 1). The new score showed a significantly higher area under curve (0.785) compared with other scores (*P* < .001).

**Conclusion:**

The early DCI score provides a practical method at the early 72 hours after aSAH to predict DCI.

## 1 INTRODUCTION

Aneurysmal subarachnoid hemorrhage (aSAH) is a serious subtype of hemorrhagic stroke carrying high mortality and morbidity.^1^, ^2^, ^3^ It is well known that delayed cerebral ischemia (DCI) plays an important role in the development of unfavorable outcomes after aSAH.^4^ The incidence of DCI is influenced by several characteristics, including demographics (such as age, sex, personal history, and past medical history),^5^ clinical status, and radiological changes at admission.^6^, ^7^ Previous studies have established several grading systems based on clinical or radiologic factors to predict incidence of DCI or outcome to guide treatment.^8^

The Glasgow Coma Scale (GCS), Hunt‐Hess (HH), and World Federation of Neurosurgical Societies (WFNS) are the widely used clinical grading scales, focusing on signs and symptoms to assess brain injury and prognosis; however, these grading systems do not take into account the volume and severity of bleeding.^8^ With the development of computed tomography (CT), some radiographic scales have been further established. By quantifying thickness and location of subarachnoid blood on CT image, the Fisher Scale (FS) and the modified Fisher Scale (mFS) predict the incidence of cerebral vasospasm and DCI.^9^ Recent Subarachnoid Hemorrhage Early Brain Edema Score (SEBES) is a new scoring system which reflects the degree of early brain injury (EBI) to predict occurrence of DCI. However, further studies are needed to determine the accuracy and effectiveness of this score.^7^, ^10^ The weakness of these radiological scales is underestimating the importance of the patients’ clinical signs. Thus, some combined grading systems were promoted, such as VASOGRADE (VG) and the HAIR scale, to predict the outcome of aSAH patients.^11^, ^12^ However, these grading systems do not consider the importance of EBI. Recent studies have demonstrated that the incidence of DCI was associated with the degree of severity of EBI after SAH.^10^

Our objective is to create a new risk score consistent with clinical and radiologic factors, and place emphasis on brain changes in the early period after SAH (within 72 hours) to predict DCI. Considering the complexity and multifactor aspects of aSAH progress, a new risk score would be established by risk stratification which integrates risk factors at early 72 hours. An efficient scoring system may provide early guidance for DCI prevention after aSAH.

## 2 METHODS

### 2.1 Study population

Our study retrospectively reviewed 1119 consecutive SAH patients admitted to our institution from January 1, 2014, to December 31, 2015. SAH was diagnosed by initial CT scan or lumbar puncture at the time of admission. Negative angiograms and arteriovenous malformation ruptures were excluded. The exclusion criteria also included the following: SAH due to trauma or suspicious trauma; patients with previous history of brain injury (such as stroke and cerebral hemorrhage, which left chronic change on the CT); SAH accompanied by serious comorbidities before SAH onset (such as severe coagulation disorders, malignant tumor, uncontrollable heart disease, and hypertension, which would interfere with clinical judgment); patients whose initial CT scan was not available for review; and patients whose initial CT was performed more than 3 days after initial presentation of SAH (in order to ensure the consistency of evaluation time of clinical and radiological data). All aspects of this study received approval from the Institutional Review Board of the Second Affiliated Hospital of Zhejiang University. Informed consent was either obtained by the patients, family members, or waived by the Institutional Review Board.

### 2.2 Variables

Demographic information, clinical, and radiological data of aSAH patients at admission were collected as the main variables. Demographic information included age (analyzed by continuous variable and categorical variable which was stratified into >40, >50, >60, and >70), sex, history of smoking, drinking, hypertension, diabetes, hyperlipidemia, and use of anticoagulants. Early brain change was quantified by clinical and radiological variables. Clinical variables included WFNS grade^13^ and HH scale.^14^ Poor clinical condition was defined as high WFNS (4‐5) and HH (4‐5). Radiological variables included intraventricular hemorrhage (IVH) and intra‐parenchymal hematoma on the initial CT scan, the mFS scale,^9^ and SEBES scale.^7^ Large amount of bleeding was defined as high mFS (3‐4); severe cerebral edema was defined as high SEBES (3‐4).

### 2.3 Outcomes

Outcomes were defined by occurrence of DCI. DCI was defined as appearing clinical vasospasm or/and delayed cerebral infarction. (a) Clinical deterioration (GCS by ≥2 points, or development of new motor deficits, which excluding other etiologies) was considered as clinical vasospasm; (b) new infarct on brain CT that was not visible on the initial CT, excluding infarctions that appeared around the aneurysm within 48 hours after aneurysm surgery or endovascular treatment, was considered as delayed cerebral infarction.^15^, ^16^ Other complications, such as rebleeding (new or expanded hemorrhage on CT), hydrocephalus, and seizures, were also recorded. All radiological data were independently and retrospectively evaluated by two blinded senior neurologists from our institution. An independent third examiner was used when there was a divergence between the two neurologists.

### 2.4 Statistical analysis

Data are presented as mean ± SD, number (percentage), odds ratio (OR), and 95% confidence interval (CI). All *P*‐values were two tailed, and a *P* < .05 was considered statistically significant. All statistical analyses were performed using SPSS 22.0 (SPSS Institute) and MedCalc Statistical Software version 18.2.1 (MedCalc Software bvba, Ostend, Belgium; http://www.medcalc.org; 2018).

For univariate analyses, continuous variables were compared between DCI patients and non‐DCI patients by using unpaired Student's *t*‐tests. Categorical variables were compared using chi‐square or Fisher's exact tests. All variables with *P* < .10 in the univariate analysis were included in the multivariate logistic regression model. Multivariate logistic regression model using backward selection was used to determine the independent predictors of DCI. Collinearity diagnosis analysis was performed to exclude the strong collinearity relation between variables before multivariate logistic regression. Multivariate logistic regression with stepwise backward selection was used to determine the independent predictors of DCI. The results of the multivariate logistic regression analysis are reported as regression coefficient (B), odd ratio (OR) at a 95% CI, and *P*‐values. Based on the predictors obtained from multivariable logistic regression, we designed a risk stratification score to predict the incidence of DCI. Each predictor was given related risk score according to the ratio of corresponding B to minimum B (Bx/Bmin) and rounding to the nearest integer, which was considered to be associated with little significant difference to the calibration and discrimination of the model.^17^

Performance of new DCI model was evaluated by assessing the calibration and discrimination. The discriminative ability of the risk score was first tested by the area under the receiver operating characteristics curve (ROC), and compared to other grading systems including HH, WFNS, mFS, and SEBES for prediction of DCI. The area under ROC curve (AUC) larger than 0.750 was considered to have good predictive accuracy.^17^ Delong test was used to compare AUC values.^18^ Calibration was assessed by the Hosmer‐Lemeshow test and calibration plot in cohorts, and *P‐*values > .05 defined good calibration.

A separate validation cohort of aSAH patients from January 2016 to April 2016 was used for internal validation of the new model. We applied the same inclusion and exclusion criteria. The performance was also evaluated by discrimination (AUC) and calibration.

## 3 RESULTS

### 3.1 Patient characteristics

**Figure 1:**
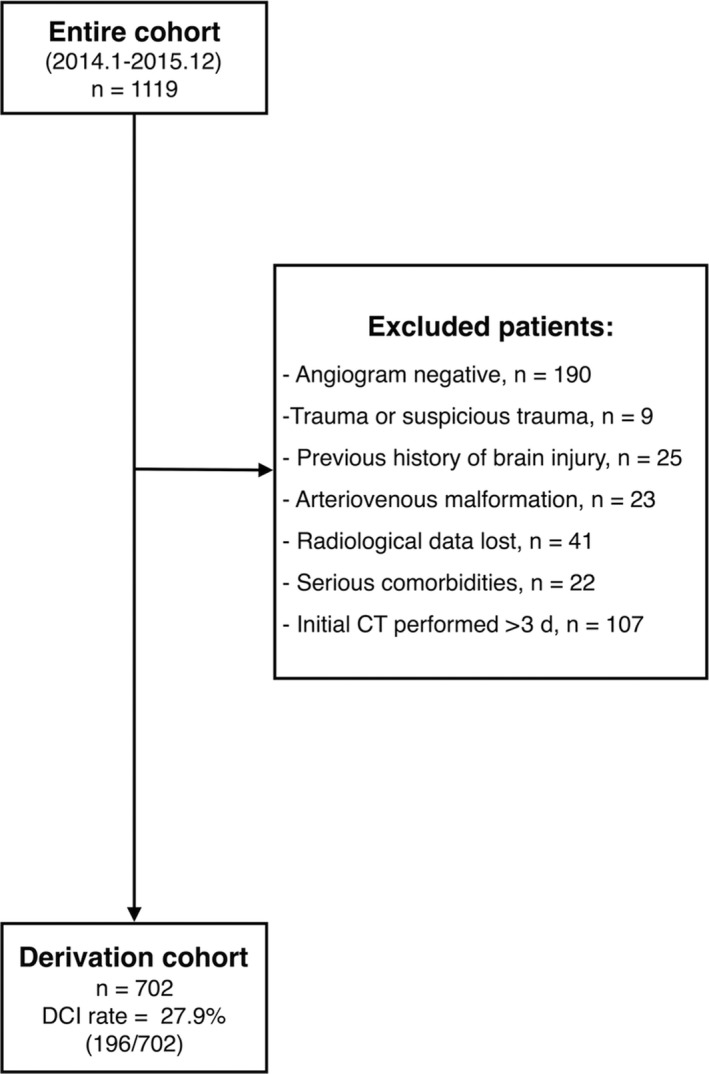
Cohort flowchart of aSAH patients*.* A total of 1119 SAH patients between 2014 and 2015 were retrospectively reviewed. After selection, 702 aSAH patients were included. aSAH: aneurysmal subarachnoid hemorrhage

In total, 702 aSAH patients were included in the cohort. The mean age was 56.0 ± 11.2, ranging from 24 to 89, and 264 (37.6%) were male. The incidence of DCI was 27.9% (196/702) in the entire cohort (Figure 1). A total of 135 (19.2%) patients suffered both clinical cerebral vasospasm and delayed cerebral infarction. Sixty‐eight (9.7%) patients suffered only clinical vasospasm, with 60 (8.5%) patients suffered only delayed cerebral infarction. Baseline characteristics are described in Table 1.

**Table 1:** Patient Characteristics

		n = 702 (%)
Male sex		264 (37.6)
Age		56.0 ± 11.2
Smoker		163 (23.2)
Drinker		202 (28.8)
Hypertension		271 (38.6)
Hyperlipidemia		247 (35.2)
Diabetes		29 (4.1)
Previous heart disease		7 (1.0)
Antiplatelet or anticoagulant		36 (5.1)
Clinical data		
GCS	13‐15	535 (76.2)
	9‐12	67 (9.5)
	3‐8	100 (14.2)
WFNS	1	477 (67.9)
	2	54 (7.7)
	3	9 (1.3)
	4	101 (14.4)
	5	61 (8.7)
HH	1	77 (11.0)
	2	385 (54.8)
	3	113 (16.1)
	4	103 (14.7)
	5	24 (3.4)
Radiological data		
mFS	0	30 (4.3)
	1	77 (11.0)
	2	151 (21.5)
	3	161 (22.9)
	4	283 (40.3)
SEBES	0	164 (23.4)
	1	81 (11.5)
	2	106 (15.1)
	3	73 (10.4)
	4	278 (39.6)
IVH		262 (37.3)
Hematoma		100 (14.2)
Aneurysm		
Anterior circulation location		418 (59.5)
Size (mm)^a^		4.5 ± 3.4
Multiple aneurysms		41 (5.8)
Treatment		
Clipping		349 (49.7)
Coiling		320 (45.6)
Others^b^		33 (4.7)
Complications		
DCI		196 (27.9)
Hydrocephalus		109 (15.5)
Rebleeding		14 (2.0)
Seizure		14 (2.0)

Abbreviations: DCI, delayed cerebral ischemia; GCS, Glasgow Coma Scale; H‐H, Hunt‐Hess; IVH, intraventricular hemorrhage; mFS, modified Fisher Scale; SEBES, Subarachnoid Hemorrhage Early Brain Edema Score; WFNS, World Federation of Neurosurgical Societies.

a Reviewed from 661 single aneurysm patients and lost data of 75 patients.

b 33 had other treatment, such as only external ventricular drainage, or only decompressive craniectomy, or bypass surgery, or refused surgery.

### 3.2 Model development

Patients who suffered DCI were prone to having higher HH (37.8% vs 10.5%), WFNS (51.5% vs 12.1%), mFS (86.7% vs 54.2%), and SEBES scores (68.4% vs 42.9%; all *P* < .001). IVH (62.2% vs 27.7%) and intra‐parenchymal hematoma (28.1% vs 8.9%; all *P* < .001) were more commonly presented in the CT images of DCI patients. The DCI patients had more aneurysms located in the anterior circulation (65.3% vs 57.3%, *P* = .011). There is no significant difference in age in continuous variables and categorical variables, despite a trend in DCI patients being older than 60 (41.3% vs 33.8%, *P* = .062; Table 2). Thus, eight predictors including age >60, high WFNS, high HH, high mFS, high SEBES, IVH, intra‐parenchymal hematoma, and aneurysms in the anterior circulation meet the criterion and were included into the multivariable logistic regression model. We excluded high HH due to the high collinearity with high WFNS (Supplemental Table S1 and S2). Finally, four predictors were retained as follows: WFNS (OR = 4.057, 95% CI = 2.627‐6.266, *P* < .001), mFS (OR = 2.623, 95% CI = 1.589‐4.331, *P* < .001), SEBES (OR = 1.539, 95% CI = 1.028‐2.305, *P* = .036), and IVH (OR = 1.932, 95% CI = 1.284‐2.906, *P* = .002). According to the regression coefficient, we assigned related scores to each predictor (Table 3). The new risk score ranged from 0 to 7. The new score was named as EDCI score, which can be used to early predict DCI.

**Table 2:** Univariate Analysis of Characteristics of SAH Patients (n = 702)

Variable	DCI (n = 196)	Non‐DCI (n = 506)	*P‐*value^a^
Risk factors			
Gender, male	82 (41.4)	182 (36.0)	.150
Age			
Mean	57.0 ± 11.1	55.6 ± 11.3	.139
>50	137 (69.9)	324 (64.0)	.142
>60	81 (41.3)	171 (33.8)	**.062**
>70	16 (8.2)	50 (9.9)	.484
Smoker	52 (26.5)	111 (21.9)	.196
Drinker	64(32.7)	138 (27.3)	.158
Hypertension	79 (40.3)	192 (37.9)	.564
Hyperlipidemia	67 (34.2)	180 (35.6)	.729
Diabetes	9 (4.6)	20 (4.0)	.703
Previous heart disease	3 (1.5)	4 (0.8)	.406
Antiplatelet or anticoagulant	11 (5.6)	25 (4.9)	.718
Clinical variable			
WFNS 4‐5	101 (51.5)	61 (12.1)	**<.001**
H‐H 4‐5	74 (37.8)	53 (10.5)	**<.001**
Radiological variable			
mFS 3‐4	170 (86.7)	274 (54.2)	**<.001**
SEBES 3‐4	134 (68.4)	217 (42.9)	**<.001**
IVH	122 (62.2)	140 (27.7)	**<.001**
Hematoma	55 (28.1)	45 (8.9)	**<.001**
Aneurysm			
Anterior circulation	128 (65.3)	290 (57.3)	**.011**
Multiple aneurysms	16 (8.2)	25 (4.9)	.102
Aneurysm size^b^	4.6 ± 3.0	4.5 ± 2.1	.538

Abbreviations: H‐H, Hunt‐Hess; IVH, intraventricular hemorrhage; mFS, modified Fisher Scale; SEBES, Subarachnoid Hemorrhage Early Brain Edema Score; WFNS, World Federation of Neurosurgical Societies.

a *P*‐values are calculated by Pearson chi‐square test or Fisher's exact test.

b Reviewed from 661 single aneurysm patients and lost data of 75 patients.

**Table 3:** Multivariate Risk Stratification Score to Predict DCI after SAH^a^

Variables	B (SE)	OR	95% CI	*P‐*value	Risk score
High WFNS	1.401 (0.222)	4.057	2.627‐6.266	<.001	3
High mFS	0.964 (0.256)	2.623	1.589‐4.331	<.001	2
High SEBES	0.431 (0.206)	1.539	1.028‐2.305	.036	1
IVH	0.658 (0.208)	1.932	1.284‐2.906	.002	1
Intercept	−2.587 (0.229)			<.001	

Abbreviations: CI, confidence interval; DCI, delayed cerebral ischemia; IVH, intraventricular hemorrhage; mFS, modified Fisher Scale; OR, odds ratio; SAH, subarachnoid hemorrhage; SE, standard error; SEBES, Subarachnoid Hemorrhage Early Brain Edema Score; WFNS, World Federation of Neurosurgical Societies.

a Data obtained from multivariable logistic regression and presented as regression coefficient (B), SE, OR, 95% CI, and *P*‐value. Risk score was derived from the B value/ 0.431 (B value of high SEBES).

### 3.3 Model performance

**Figure 2:**
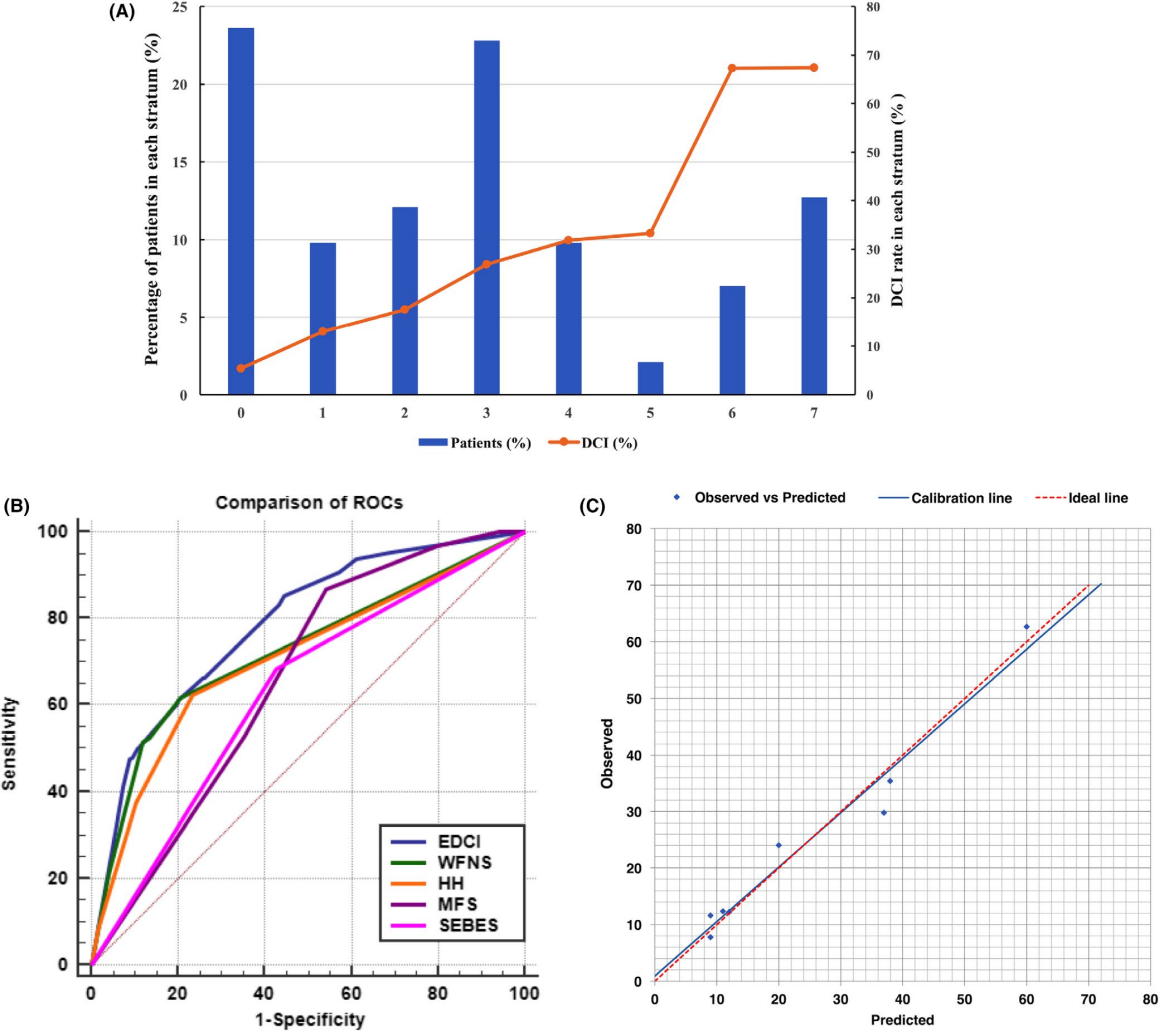
A, DCI rate based on EDCI score. Distribution of the EDCI score (dark blue bars) and corresponding observed DCI rate (orange points) for each score. B, ROC of EDCI score and other grading systems. EDCI score keeps a highest AUC (AUC = 0.785, 95% CI = 0.752‐0.815) among these grading systems (AUC_WFNS_ = 0.724, 95% CI = 0.689‐0.757; AUC_HH_ = 0.706, 95% CI = 0.671‐0.739; AUC_SEBES_ = 0.660, 95% CI = 0.624‐0.695; AUC_mFS_ = 0.627, 95% CI = 0.624‐0.695). *P* value was < .001 compared to each score. C, Calibration plot for predicted versus observed DCI for the risk EDCI score. Calibration plot, *P* = .522. AUC, area under receiver operating characteristics curve; CI, confidence interval; DCI, delayed cerebral ischemia; HH, Hunt‐Hess; mFS, modified Fisher Scale; ROC, receiver operating characteristics curve; SEBES, Subarachnoid Hemorrhage Early Brain Edema Score; WFNS, World Federation of Neurosurgical Societies

The distribution of the EDCI score is shown in Figure 2A. The score increase was associated with an increase in the DCI rate (*P* < .001 for trend). The positive predictive values for each score are shown in Table 4. Scores ≤1 are associated with low risk of DCI (OR as reference), scores ranging from 2 to 4 are associated with moderate risk (OR = 4.122, 95% CI 2.393‐7.099), and scores ≥5 are associated with high risk (OR = 21.481, 95% CI = 11.988‐38.490).

**Table 4:** Related DCI risk to each risk point

Risk point	Estimated DCI risk	Risk group	OR	95% CI
0	7.0	Low	Reference	
1	11.0			
2	17.0	Moderate	4.122	2.393‐7.099
3	25.2			
4	35.7			
5	47.8	High	21.481	11.988‐38.490
6	60.2			
7	71.4			

Abbreviations: CI, confidence interval; DCI, delayed cerebral ischemia; OR, odds ratio.

The discriminative ability of the EDCI risk score was good in the ROC (AUC = 0.785, 95% CI = 0.752‐0.815; Figure 2B). The AUC was significantly higher compared with clinical and radiological scores. The EDCI score has the highest AUC among these grading systems (AUC_WFNS_ = 0.724, AUC_HH_ = 0.706, AUC_SEBES_ = 0.660, AUC_mFS_ = 0.627). The calibration ability was also good in Hosmer‐Lemeshow test (*P* > .05; Figure 2C).

A total of 108 patients were included in our internal validation analysis. The baseline characteristics are described in Supplemental Table S3. The AUC was 0.773 (95% CI = 0.683 to 0.848) for predicting DCI. The outcome was systematically similar to our predictions (*P* > .05 in Hosmer‐Lemeshow test) (Figure S1).

## 4 DISCUSSION

In this study, we identified a risk stratification model based on the early variables acquired within 72 hours after aSAH to predict the development of DCI. We named this new risk score “EDCI” score, which is comprised of four early independent predictors: WFNS, mFS, SEBES, and IVH. These four factors are clinically intuitive and easily assessed by practitioners and neurosurgeons at admission. The EDCI score successfully combined the clinical and radiological risk factors, avoiding the one‐sidedness of sole grading systems, and reflects the early brain change after aSAH. The higher AUC indicated that EDCI performed better in the prediction of DCI compared with other scores.

Cerebral injury within the first 72 hours after hemorrhage plays an important role in the progress of complications and outcomes after aSAH.^10^ Generally, acute injury after aSAH can be indirectly reflected by the clinical and radiological data. SEBES, which is considered an indicator of EBI, qualitatively assesses the degree of global cerebral edema after SAH. High‐grade SEBES is an indicator of an increased risk of developing DCI after hemorrhage.^7^ Another radiologic factor, “mFS,” based on quantifying the amount of blood quite possibly influences the degree of EBI and predicts CVS and DCI.^9^ Large evidence proved higher mFS was a significant risk factor for DCI.^12^, ^19^, ^20^ Additionally, among the aSAH patients, a higher WFNS score at admission was considered a risk factor for DCI.^7^, ^20^, ^21^ The “WFNS” factor is derived from GCS score and focuses on signs and symptoms of SAH patients to reflect the primary brain injury at admission.^8^ It is widely applied in many combined scores, such as VG^12^ and modified WFNS.^22^

Improving the FS, the mFS places greater emphasis on ventricular blood. Claassen et al^23^ affirmed that IVH in lateral ventricles is an independent risk factor for the development of DCI (OR = 4.1, 95% CI = 1.7‐9.8). Using the presence of bilateral IVH, they divided thin SAH into grades 0‐1 and 2, thick SAH into grades 3 and 4.^24^ The limitation was that large unilateral IVH was regarded as no IVH by mFS, which may weaken the influence of unilateral IVH.^25^ Furthermore, in our study, we divided grades 0‐2 and 3‐4 into the same grade for easy record, which potentially weakens the importance of IVH. Thus, we added the IVH into the risk factors, and there was no collinearity between mFS and IVH. IVH also showed its value from multivariate analysis, which was similar in the HAIR score.^11^

It should be mentioned that age, an important risk factor, was excluded from our score. The study has tested the age by continuous variable and categorical variable in univariate analysis. However, consistent with a recent promoted DCI risk score, there was no statistical significance found in our study.^26^ The current studies on the prognostic value of age are controversial. Some studies suggested that older age is associated with lower incidence of DCI,^24^ and another study suggested aging patients were more likely to suffer DCI compared with younger patients.^19^ Interestingly, also other studies found that aging has no difference in the incidence of DCI.^27^, ^28^ This divergence may derive from the differences in the division of age groups in each study. Older age is associated with larger subarachnoid clot volume, which is considered to lead to DCI.^29^ Conversely, another hypothesis was the cerebral vessels are stiffer (caused by atherosclerosis, collagen fiber increase etc.) in elderly patients, making them more resistant to vasospasms.^26^

The current SAH grading system can be divided into three groups: clinical, radiological, and combined grading systems.^15^ The strength of a single clinical and radiological scale is its convenience to predict relevant complications and outcomes. Many clinical grading scales, such as GCS, WFNS, and HH, were initially designed for the prediction of prognosis and treatment selection in different neurological diseases (traumatic brain injury, aSAH etc.). The primary purpose of these clinical scales is for effective communication among clinicians. However, these clinical scores may potentially underestimate risks for conscious patients without severe neurological deficits who have thick subarachnoid blood clots or IVH.^15^ Although the radiologic scales such as mFS are developed to predict the incidence of vasospasm or DCI, it underestimates the importance of IVH or intra‐parenchymal hematoma as we previously mentioned.^25^ Subsequently, some investigators promote combined scores to overcome the limitations of separate clinical and radiological scores.^11^, ^12^ HAIR used a practical method of risk stratification to find the independent risk factors to predict the in‐hospital mortality of SAH patients.^11^ VG simply combined mFS and WFNS into three categories: green, yellow and red, which distinguished patients between good WFNS grades, with and without significant SAH regarding the prediction of DCI.^12^ Although VG was developed by two early predictors, and available for risk stratification at the time after SAH, the AUC was not large according to results from recent study.^15^ Dengler et al^15^ retrospectively reviewed 423 consecutive patients with aSAH and showed that the combined scores had no superiority to the single grading system in predicting new cerebral infarction (mFS 0.612, WFNS 0.672, H‐H 0.673, VG 0.674, HAIR 0.638).

Other risk stratification scores usually have a large range of scores, which may inconvenience practitioners and limit their applicability.^17^, ^26^, ^30^, ^31^ Unlike other risk stratification scores, we use a dichotomy to divide risk factors into categorical variables (eg, high WFNS and low WFNS), and the variables were only limited to those available within 72 hours after aSAH. This makes our score more convenient to use. Besides, several new methods were promoted to predict the DCI, which may provide us a new way to measure the early brain changes after aSAH, such as using permeability imaging, intracerebral probes, and multimodal neuromonitoring.^32^, ^33^, ^34^ However, the relatively small sample size and relative inconvenient variable acquisition of this study limited their reliability and practicality. Prospective designs are needed for future validation. Furthermore, another model focuses on murine retina to detect transient impairment of transretinal signaling by unconjugated bilirubin (an organotypical neuronal network from wild type), which was considered as a related indicator for development of vasospasm and DCI after aSAH. This also contributed another new way to measure early brain changes after aSAH.^35^

### 4.1 Limitations

Despite the strengths of our new risk score for prediction DCI, this study presents some limitations. First, our study is based on a single‐center retrospective observational study. There would be some potential biases and known and unknown confounders in this study. The patients with WFNS 4‐5 (23.1%) and mFS 3‐4 (63.3%) were consistent with other studies (WFNS: 22.4%‐31.1%^12^, ^26^, ^36^; mFS: 56.2%‐77.7%^20^, ^26^, ^36^). Additionally, all CT grades were evaluated by two blinded senior neurologists. The DCI rates in our study were 27.9% in the cohort, which is consistent with other studies (21.0%‐31.2%^7^, ^12^, ^16^, ^20^, ^21^, ^26^). To limit the potential confounders, we excluded patients whose SAH onset was more than 3 days before obtaining a CT scan to ensure the timing of the evaluation of each variable. We also excluded patients with serious comorbidities who may have had cerebrovascular events before SAH onset, as this may potentially interfere with clinical judgment. However, this may also result this manuscript cannot be generalized to total population of SAH patients. Second, an unequal distribution of patients in each stratum may be the common limitation of risk score.^30^, ^37^ This may be caused by the different score assignment of each variable. Third, despite validation of the EDCI score performance, it was not yet complete. The DCI rate for stratum 5‐7 appears to abruptly change in the derivation cohort of our study. All in all, future studies need to use a larger external validation cohort to reassess performance of EDCI score in DCI prediction.

## 5 CONCLUSION

In summary, the EDCI score is a robust tool for quickly predicting the early incidence of DCI after aSAH. It obtains the risk factors available with 72 hours after aSAH and reflects the early brain change after aSAH. The EDCI score presents very good discriminative and calibration properties. Neurosurgeons may use this risk score to guide DCI prevention for aSAH patients.

## CONFLICT OF INTEREST

The authors declare that they have no conflict of interest.
