# Supplementary figures and images for: New risk score of the early period after spontaneous subarachnoid hemorrhage: For the prediction of delayed cerebral ischemia
Source: CNS Neurosci Ther. 2019 Aug 12;25(10):1173–81. doi: 10.1111/cns.13202 (PMC6776741; doi:10.1111/cns.13202)

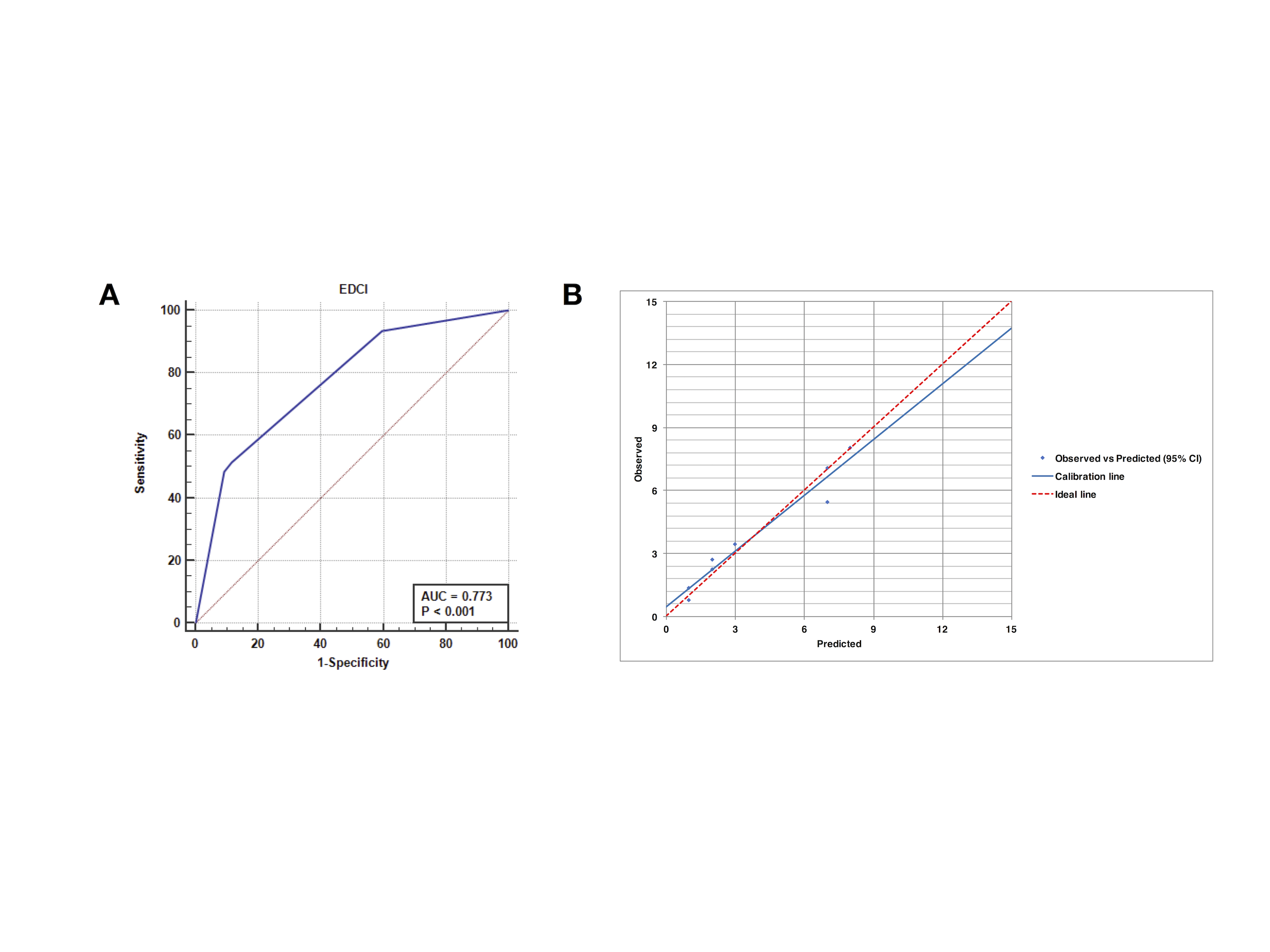

Supplement: Supplementary file 1 [file CNS-25-1173-s001.tiff]
