# Supplementary material for: New risk score of the early period after spontaneous subarachnoid hemorrhage: For the prediction of delayed cerebral ischemia
Source: CNS Neurosci Ther. 2019 Aug 12;25(10):1173–81. doi: 10.1111/cns.13202 (PMC6776741; doi:10.1111/cns.13202)
Supplement: Supplementary file 2 [file CNS-25-1173-s002.docx]

| **Supplemental Table S1.** **Collinear statistics** | | |
| --- | --- | --- |
| **Variables** | **Tolerance** | **VIF** |
| **Age > 60** | 0.884 | 1.131 |
| **High WFNS** | 0.322 | 3.106 |
| **High HH** | 0.343 | 2.914 |
| **High mFS** | 0.696 | 1.437 |
| **High SEBES** | 0.767 | 1.304 |
| **IVH** | 0.736 | 1.358 |
| **Hematoma** | 0.821 | 1.218 |
| **Anterior circulation** | 0.947 | 1.056 |
| **Abbreviations:** VIF, Variance Inflation Factor; H-H, Hunt-Hess; IVH, intraventricular hemorrhage; mFS, modified Fisher Scale; SEBES, Subarachnoid Hemorrhage Early Brain Edema Score; WFNS, world federation of neurosurgical societies | | |

| **Supplemental Table S2. Collinear statistics** | | | | | | | | | | | |
| --- | --- | --- | --- | --- | --- | --- | --- | --- | --- | --- | --- |
| **Dimension** | **Eigenvalue** | **Condition Index** | **Constant** | **Age > 60** | **High WFNS** | **High HH** | **High mFS** | **High SEBES** | **IVH** | **Hematoma** | **Anterior circulation** |
| 1 | 5.149 | 1 | 0.01 | 0.01 | 0.01 | 0.01 | 0.01 | 0.01 | 0.01 | 0.01 | 0.01 |
| 2 | 1.14 | 2.125 | 0.02 | 0.02 | 0.05 | 0.07 | 0.01 | 0.01 | 0.01 | 0.05 | 0.05 |
| 3 | 0.741 | 2.636 | < 0.001 | 0.41 | 0.01 | 0.01 | < 0.001 | 0.05 | 0.01 | 0.24 | 0.02 |
| 4 | 0.636 | 2.846 | < 0.001 | 0.17 | 0.01 | 0.03 | < 0.001 | 0.06 | < 0.001 | 0.65 | 0.01 |
| 5 | 0.436 | 3.436 | < 0.001 | < 0.001 | 0.02 | 0.04 | 0.01 | 0.15 | 0.36 | < 0.001 | 0.32 |
| 6 | 0.392 | 3.625 | < 0.001 | 0.09 | 0.01 | < 0.001 | 0.02 | 0.24 | 0.59 | 0.01 | 0.14 |
| 7 | 0.206 | 5.002 | 0.01 | 0.07 | < 0.001 | < 0.001 | 0.93 | 0.33 | 0.01 | 0.02 | 0.04 |
| 8 | 0.15 | 5.849 | < 0.001 | 0.02 | **0.89** | **0.83** | < 0.001 | 0.01 | < 0.001 | 0.01 | < 0.001 |
| 9 | 0.15 | 5.863 | 0.95 | 0.21 | < 0.001 | < 0.001 | 0.02 | 0.15 | 0.01 | 0.01 | 0.41 |
| **Abbreviations:** H-H, Hunt-Hess; IVH, intraventricular hemorrhage; mFS, modified Fisher Scale; SEBES, Subarachnoid Hemorrhage Early Brain Edema Score; WFNS, world federation of neurosurgical societies, | | | | | | | | | | | |

| **Supplemental Table S3. Patient characteristics of validation cohort** | | |
| --- | --- | --- |
|  |  | n = 108 (%) |
| Male sex |  | 44(40.7) |
| Age |  | 56.1 ± 11.3 |
| Smoker |  | 26(24.1) |
| Drinker |  | 19(17.6) |
| Hypertension |  | 39(38.6) |
| Hyperlipidemia |  | 247(36.1) |
| Diabetes |  | 5(4.6) |
| Previous heart disease |  | 1(0.9) |
| Antiplatelet or anticoagulant |  | 0(0) |
| **Clinical data** |  |  |
| GCS | 13-15 | 83(76.9) |
|  | 9-12 | 12(11.1) |
|  | 3-8 | 13(12.0) |
| WFNS | 1 | 66(61.1) |
|  | 2 | 10(9.3) |
|  | 3 | 7(6.5) |
|  | 4 | 20(18.5) |
|  | 5 | 5(4.6) |
| HH | 1 | 14(13.0) |
|  | 2 | 59(54.6) |
|  | 3 | 16(14.8) |
|  | 4 | 16(14.8) |
|  | 5 | 3(2.8) |
| **Radiological data** |  |  |
| mFS | 0 | 4(3.7) |
|  | 1 | 11(10.2) |
|  | 2 | 21(19.4) |
|  | 3 | 23(21.3) |
|  | 4 | 49(45.4) |
| SEBES | 0 | 33(30.6) |
|  | 1 | 7(6.5) |
|  | 2 | 14(13.0) |
|  | 3 | 9(8.3) |
|  | 4 | 45(41.7) |
| IVH |  | 36(33.3) |
| Aneurysm |  |  |
| Anterior circulation Location |  | 68(63.0) |
| Size (mm) ^‡^ |  | 4.5 ± 2.0 |
| Multiple aneurysms |  | 6(5.6) |
| **Treatment** |  |  |
| Clipping |  | 62(57.4) |
| Coiling |  | 46(42.6) |
| **Complications** |  |  |
| DCI |  | 31(28.7) |
| Hydrocephalus |  | 18(16.7) |
| Rebleeding |  | 3(2.8) |
| Seizure |  | 4(3.7) |
| ^‡^Reviewed from 102 single aneurysm patients and lost data of 13 patients;  Abbreviations: DCI: delayed cerebral ischemia; GCS, Glasgow Coma Scale; H-H, Hunt-Hess; IVH, intraventricular hemorrhage; mFS, modified Fisher Scale; SEBES, Subarachnoid Hemorrhage Early Brain Edema Score; WFNS, world federation of neurosurgical societies. | | |
